# Calcium Sulfate Disks for Sustained-Release of Amoxicillin and Moxifloxacin for the Treatment of Osteomyelitis

**DOI:** 10.3390/ma17164086

**Published:** 2024-08-17

**Authors:** Riddhi Gangolli, Smruti Pushalkar, Bryan G. Beutel, Natalie Danna, Simone Duarte, John L. Ricci, Kenneth Fleisher, Deepak Saxena, Paulo G. Coelho, Lukasz Witek, Nick Tovar

**Affiliations:** 1 Biomaterials Division, NYU Dentistry, New York, NY 10010, USAbryanbeutel@gmail.com (B.G.B.); lukasz.witek@nyu.edu (L.W.); 2 Center for Genomics and Systems Biology, Department of Biology, New York University, New York, NY 10003, USA; 3 Department of Restorative Dentistry, University at Buffalo School of Dental Medicine, Buffalo, NY 14215, USA; 4 Department of Oral and Maxillofacial Surgery, NYU Dentistry, New York, NY 10010, USA; 5 Department of Molecular Pathobiology, NYU Dentistry, New York, NY 10010, USA; ds100@nyu.edu; 6 DeWitt Daughtry Family Department of Surgery, Division of Plastic Surgery, Miller School of Medicine, University of Miami, Miami, FL 33146, USA; pgc51@med.miami.edu; 7 Department of Biochemistry and Molecular Biology, Miller School of Medicine, University of Miami, Miami, FL 33146, USA; 8 Hansjörg Wyss Department of Plastic Surgery, NYU Grossman School of Medicine, New York, NY 10016, USA; 9 Department of Biomedical Engineering, NYU Tandon School of Engineering, Brooklyn, NY 11201, USA

**Keywords:** calcium sulfate, infection, antibiotic, osteomyelitis, drug release

## Abstract

The purpose of this in vitro study was to develop calcium sulfate (CS)-based disks infused with an antimicrobial drug, which can be used as a post-surgical treatment modality for osteomyelitis. CS powder was embedded with 10% antibiotic, amoxicillin (AMX) or moxifloxacin (MFX), to form composite disks 11 mm in diameter that were tested for their degradation and antibiotic release profiles. For the disk degradation study portion, the single drug-loaded disks were placed in individual meshes, subsequently submerged in phosphate-buffered saline (PBS), and incubated at 37 °C. The disks were weighed once every seven days and analyzed via Fourier-transform infrared spectroscopy, X-ray diffraction, energy dispersive X-ray spectroscopy, and scanning electron microscopy. During the antibiotic release analysis, composite disks were placed in PBS solution, which was changed every 3 days, and analyzed for antibiotic activity and efficacy. The antibacterial effects of these sustained-release composites were tested by agar diffusion assay using *Streptococcus mutans* (*S. mutans*) UA 159 as an indicator strain. The degradation data showed significant increases in the degradation of all disks with the addition of antibiotics. Following PBS incubation, there were significant increases in the amount of phosphate and decreases in the amount of sulfate. The agar diffusion assay demonstrated that the released concentrations of the respective antibiotics from the disks were significantly higher than the minimum inhibitory concentration exhibited against *S. mutans* over a 2–3-week period. In conclusion, CS-antibiotic composite disks can potentially serve as a resorbable, osteoconductive, and antibacterial therapy in the treatment of bone defects and osteomyelitis.

## 1. Introduction

Osteomyelitis is an infectious bone disease that remains a challenge for both diagnosis and management. It impacts the structure and viability of all components of bone. Treatment options, surgical and/or antibiotics, are based upon the individual extent of the disease and capacity to heal; often, conservative management leads to recurrence [1,2,3]. Whether acute or chronic in nature, hematogenously seeded or locally-introduced, osteomyelitis that is not treated effectively can lead to osteonecrosis and subsequent loss of function and disability. Despite relatively standard surgical approaches and a wide variety of antibiotic choices, the clinical course may lead to significant morbidity [4,5]. Current management of osteomyelitis requires parenteral antibiotics, which when administered in high doses could potentially lead to antibiotic tolerance and increased risk of side effects [6,7]. In an infected state, however, bone vasculature is typically compromised and biofilms may form on the osseous surface, making it difficult for systemic antibiotics to reach their target site in the same manner as a direct localized approach [8].

Furthermore, osteomyelitis is not limited only to long bones, but the condition may involve the mandible, maxilla, and/or nasal bones [9,10,11]. While it has been found to affect the craniofacial region, studies have indicated that the maxilla is less commonly involved than the mandible due to its highly vascular nature [10,12]. Mandibular osteomyelitis is commonly linked with dental infections, (i.e., periodontitis or abscesses), and in certain scenarios may be a result of trauma or surgery. While less common, maxillary osteomyelitis is associated with infections originating from sinus or dental infections and, in some severe cases, system conditions. A recent study reported that osteomyelitis, independent of the jaw, was more common in male patients than female patients [10].

The potential benefit for local antibiotic delivery systems is the direct delivery of a high concentration of antibiotic to a precise area, decreasing treatment times, systemic toxicity, and cost [5,13]. Furthermore, they may be used in combination with bone graft materials to fit and fill dead space while providing infection control [14,15,16]. While nonresorbable and resorbable antibiotic implant carriers, such as polymethyl methacrylate and polylactide-polyglycolide copolymers, have been studied, their disadvantages include a secondary invasive procedure to retrieve the biomaterial, foreign body reaction, variable antibiotic elution, and susceptibility to antibiotic resistance [13,17,18]. Among the ceramic-based carrier systems, calcium phosphate (CaP), tricalcium phosphate, hydroxyapatite, and calcium sulfate (CS) have been studied [14,19,20]. These ceramics are available in large quantities and, unlike autogenous and allogeneic graft materials, are reproducible, easily sterilized, and have recently been studied as carriers for stem cells, growth factors, and antibiotics [14,20,21,22,23]. Consequently, bioactive resorbable ceramic and polymeric-based carriers are currently being developed to bridge the gap between void filling and infection control. The attractive features of such a composite material include localized antibiotic delivery, well controlled drug release rate, negligible effect on drug activity, targeted drug delivery, reproducibility, and the osteogenic properties of the bioactive carrier [14,21,22,23,24,25].

Among these bioactive carriers, CS bone grafts have a well-documented and long history of use as fillers in bone defects due to their resorbable and osteoconductive properties [26,27]. In a multicenter study of patients with bone defects treated with a CS bone graft substitute, radiographic evaluation revealed that 99% of CS had been resorbed and 88% of the defect was filled with trabecular bone by 6 months [28]. Additional studies have shown that CS, when added to other graft materials, serves as an effective adjunct to enhance osteogenesis in bone defects [29]. In order to augment its robustness, the concept of embedding antibiotics into CS stems from the prevalence of secondary infection of implanted grafts and the impaired blood supply caused by microbial infection, which impedes effective systemic therapy.

Microbial infection is one of the critical causes of rejection of biomaterials as a result of adherence and colonization on any environmental surfaces. The aim of this study was to develop and characterize a synthetic bioactive graft material that is bioresorbable, osteoconductive, and combats local infection. CS was combined with amoxicillin (AMX) or moxifloxacin (MFX) to form a composite antibiotic delivery system. *Streptococcus mutans* (*S. mutans*) UA 159, the most common microorganism found in the oral cavity that can grow both aerobically and anaerobically, was used as an indicator strain to test the sustained release of these antibiotic-loaded ceramic disks. Moreover, this microbe, in abundance, can create an acidic environment, initiating demineralization of enamel, dentin, and cementum, as well as corroding restorative dental materials [30]. While the predominant microbe implicated in orthopedic osteomyelitis is *Staphylococcus aureus*, [7] *S. mutans* has been reported to be involved in vertebral osteomyelitis [31]. Therefore, the methods and results of this study would have significant application for the study and treatment of all types of osteomyelitis. Previous orthopedic-based studies have used a combination of calcium sulfate and tobramycin for the treatment of osteomyelitis with varying success [32,33].

This study evaluated and compared the degradation rate, potential osteoconductivity, and antimicrobial effect of a novel antibiotic-loaded ceramic system. CS disks coupled with either AMX or MXF were compared to a plain CS disk, the control group. Weight reduction, CaP formation, and their respective antibiotic release profiles were documented.

## 2. Materials and Methods

### 2.1. Composite Disk Production for Antibiotic Release Analysis

Antibiotic-loaded CS disks measuring 11 mm in diameter with 3 mm height were prepared using a combination of hemihydrate CS (JT Baker Chemical Company, Phillipsburg, NJ, USA) and 10% weight of either amoxicillin or moxifloxacin (Tecoland Corp., Edison, NJ, USA). CS-antibiotic composites were generated by mixing 225 mg of CS powder and 25 mg of antibiotic powder. The control CS disks were fabricated without antibiotic using 250 mg of CS powder. This mixture was then reconstituted at a ratio of 1 mg of CS to 0.3 mL of saline (weight/volume ratio) for molding the materials into the desired disk. The composite disks were classified into three groups (n = 7 per group): CS with amoxicillin (CS-AMX), CS with moxifloxacin (CS-MFX), and CS without antibiotic (CS).

### 2.2. In Vitro Degradation Rate Analysis

The disks (n = 5 per group) were separately weighed and placed individually in a nylon mesh with a pore size opening of 60 µm and 35% open area (Small Parts, Miramar, FL, USA) and tied with dental floss to form nylon bags. The nylon bags were subsequently weighed to determine the weight of the nylon mesh and floss by subtracting the disk weight from that of the nylon bags. The baseline initial weight of the disks for all ensuing calculations began after a 24 h soak in 5 mL of phosphate-buffered saline (PBS; 1× solution contains 0.137 M NaCl, 0.0027 M KCl, and 0.0119 M phosphates) in a 15 mL centrifuge tube incubated at 37 °C following blot drying. Subsequent weighing of the nylon bags was performed once every seven days after blot drying. Degradation rates were then evaluated by comparing weight changes to the initial baseline weight and continued over time until the percentage degradation reached 20%. The PBS was changed once every three days for all disks in order to prevent calcium saturation and to simulate the body’s natural ion exchange.

### 2.3. CS Surface Characterization

Disks (n = 2) from each group were reserved for surface characterization at 30 days (n = 1) and 60 days (n = 1) to assess for the presence of calcium and phosphate. Surface characterization was performed before and after incubation via three different modalities.

Fourier-transform infrared spectroscopy (FTIR, Nicolet FTIR Spectrometer, Thermo scientific, Newington, NH, USA) was performed to identify the functional groups present. Specifically, this characterization was performed to determine if there was any conversion of CS to CaP (apatite) during incubation in PBS. The CS-AMX, CS-MFX, and CS control disks were characterized using FTIR before immersion in PBS. The spectra of the samples were compared to that of synthetic bone mineral (SBM) [34]. Characterization was performed with the background of a 250 mg potassium bromide (KBr) disk. The sample disks were prepared by combining 250 mg of KBr with 1 mg of composite material (mixed with mortar and pestle). Disks were formed in the FTIR pellet press (PIKE CrushIR, Watertown, WI, USA) at 5 tons and vacuumed for 60 s. The FTIR was set to run at 256 cycles, with a resolution of 4.0 and aperture of 100.0 for both the background and sample.

Energy dispersive X-ray spectroscopy (EDS, EMItech K350, Fall River, MA, USA) was used to observe the conversion of CS to CaP and hydroxyapatite, while scanning electron microscopy (SEM, Hitachi 3500, Pleasanton, CA, USA) was used at various magnifications to observe the surface topography. Disk samples before and after immersion in PBS at 37 °C were first carbon-coated for 2 s at 5 amps in a sputter coater (EMItech K350, Fall River, MA, USA) with a carbon coater power source (EMItech CA7625, Fall River, MA, USA) for SEM imaging. The imaging was performed at 1000× magnification to determine the elemental map of the surface and the appearance of phosphate. Magnification at 10 µm and 20 µm was performed to analyze the topographical differences on the surface, which could be suggestive of apatite. EDS scans were run for 60 s and only those with counts greater than 1500 counts/s were included. Sulfate crystals have more angular and needle-shaped morphology, whereas phosphate crystals are more globular-shaped. Thus, there is a notable difference in the morphological features of these two crystals. Once the particle was identified through EDS probing, SEM recorded the morphology of the particle at a 1000× magnification.

### 2.4. Antibacterial Activity by Agar Diffusion Assay

In vitro antimicrobial activity of the single drug-loaded disks and the CS controls were tested against *S. mutans* UA 159 by agar diffusion assay for their ability to inhibit microbial growth in response to the antibiotic drug released from the CS disks. The minimum inhibitory concentration (MIC) against *S. mutans* for amoxicillin is 0.03–0.50 µg/mL [35,36] and for moxifloxacin, it is 0.12–0.25 µg/mL [36], by tube dilution method. *S. mutans* culture was maintained on Brain Heart Infusion (BHI) Agar plates (Becton Dickinson, Franklin Lakes, NJ, USA) by sub-culturing every 48 h at 37 °C in a 5% CO_2_ atmosphere. The single drug-released antimicrobial activity was analyzed over time from each of the CS-AMX, CS-MFX, or CS control disks (n = 3) for the desorbed active ingredient in PBS solution (eluent). Approximately 300 µL of PBS eluent was removed from the predetermined samples every 3 days for the period of the degradation study (20% of the original disk weight). The PBS eluent (~100 µL) was aliquoted separately into three sterile 1.5 mL Eppendorf tubes, gamma-sterilized at ~25 kGy for 45 min (Gammacell 1000 Elite, Best Theratronics, Ontario, CA, USA), and stored at −80 °C until further use.

For the agar diffusion assay, the bacterial inoculum was prepared by inoculating isolated colonies in 10 mL of sterile BHI broth (Becton Dickinson, Franklin Lakes, NJ, USA) and incubation overnight for 16–18 h under the same growth conditions. The bacterial suspension was adjusted spectrophotometrically (Biomate 3, Thermo Electron Corporation, Cincinnati, OH, USA) to an optical density (OD) of 0.3–0.35 at 660 nm to achieve approximately 10^8^ cfu/mL. The seed agar was prepared by mixing 1000 µL of bacterial suspension in 25 mL of BHI agar at 45 °C, and 4 mL was poured onto a previously set basal layer of BHI agar in 90 mm petri plates [37]. Subsequently, after cooling, three wells were bored onto each plate using a sterile stainless-steel borer (4.8 mm inner diameter). Each of the three wells was inoculated with test PBS eluent from the drug-loaded and control disks, single-drug standard, low concentration (LC) and single-drug standard, high concentration (HC), respectively. The LCs that were set for both AMX and MXF were 4–5 times higher than their minimum inhibitory concentrations (MICs) against *S. mutans* [35]. Predetermined standard LCs (10 µg/mL for AMX and 5 µg/mL for MFX in PBS) and HCs (250 µg/mL for both AMX and MFX in PBS) were used in each test plate to exclude variations between plates during the assay. The plates were maintained for 30 min at room temperature to allow for diffusion and incubated for 24 to 48 h at 37 °C in a 5% CO_2_ incubator.

Following incubation, the diameters of the zone of inhibition were measured using a caliper by considering the distance (mm) from edge to edge across the zone of inhibition over the center of the well. The correction factor based on the relative zone size of the single-drug standard compared to the mean zone size of the single-drug standard across all plates was used to normalize the individual plate responses. Experiments were performed using three elutes per samples from 2 individual experiments. Inhibition zone diameters were assessed, and the mean was calculated. Confluent bacterial growth with no zone of inhibition was observed with CS control eluents.

The standard curves for each antibiotic were derived based on inhibition zone diameters using different concentrations of each antibiotic standard (AMX or MFX) and a pre-determined inoculum concentration.

### 2.5. Statistical Analysis

Mixed-model ANOVA testing for the degradation assessments, antibiotic activity, and release investigations were performed using SPSS software (version 19.0.1, IBM, Chicago, IL, USA). All *p*-values are two-tailed, with *p* < 0.05 indicating significance.

## 3. Results

### 3.1. In Vitro Degradation Rate Analysis

**Figure 1:**
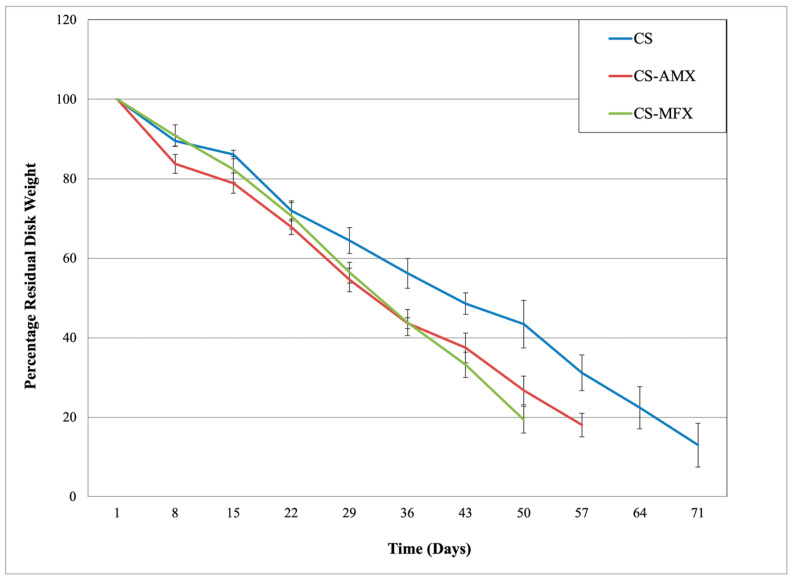
Degradation profile for calcium sulfate disks, CS controls (CS, in blue), and antibiotic-loaded composite disks with amoxicillin (CS-AMX, in red) and moxifloxacin (CS-MFX, in green).

The CS-AMX and CS-MFX disks showed some yellowing effect due to the embedded antibiotic. The addition of AMX and MFX to CS significantly increased the degradation rates of the single drug-loaded CS disks as compared to the CS control disks (*p* = 0.003). On average, the CS control disks degraded at a rate of 8.7 ± 3.1% per week, CS-AMX disks at 10.2 ± 3.7% per week, and CS-MFX disks at 11.5 ± 2.2% per week (Figure 1). Additionally, no significant difference was observed between the degradation profiles of the CS-AMX and CS-MFX groups (*p* = 0.584).

### 3.2. CS Surface Characterization

**Figure 2:**
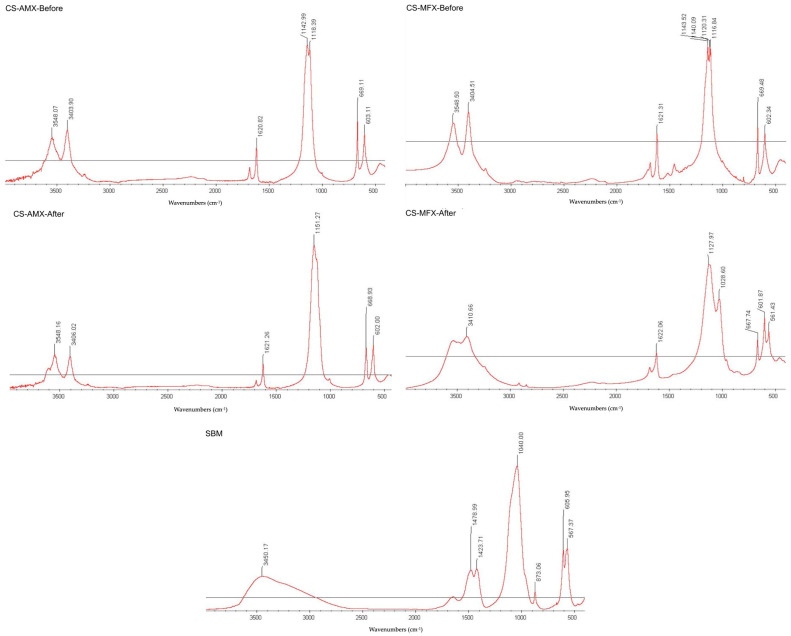
FTIR profiles before and after incubation for CS-AMX-Before and CS-AMX-After, CS-MFX-Before and CS-MFX-After. For comparison reasons SBM without incubation (**bottom**).

FTIR could not detect the presence of phosphate groups in the CS-AMX or CS-MFX disks following immersion in PBS (Figure 2). The results were compared to the FTIR spectra of the CS control group and multiple standards, as well as a synthetic bone mineral (SBM) preparation, which consisted of a carbonate apatite matrix incorporating magnesium, zinc, and fluoride ions [38].

**Figure 3:**
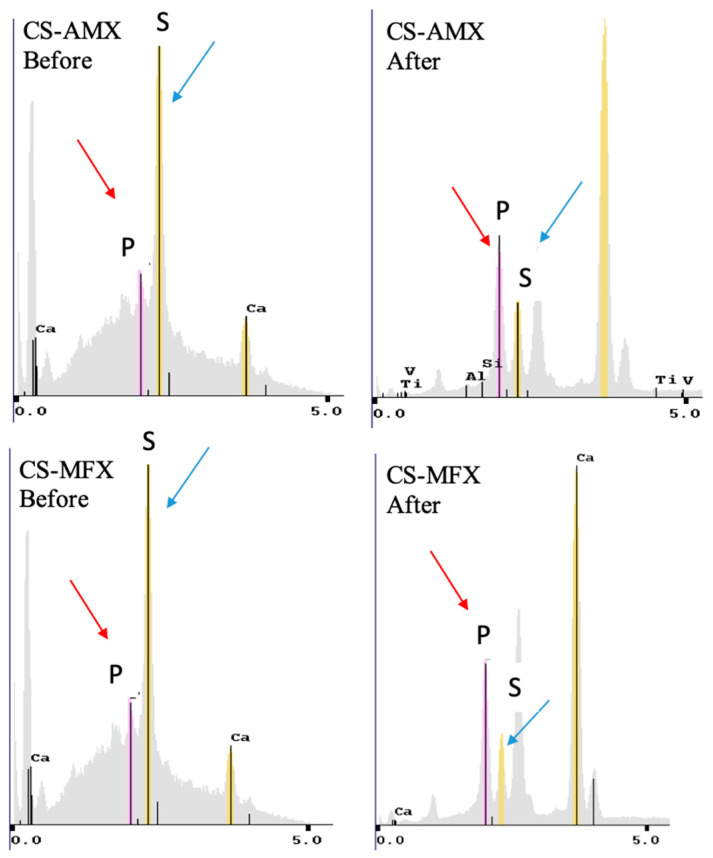
X-ray diffraction patterns comparing CS control, CS-AMX, and CS-MFX disks after immersion in PBS for 30 days.

**Figure 4:**
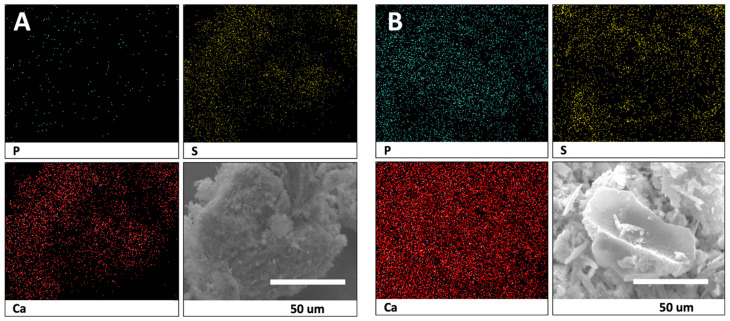
Energy dispersive X-ray spectroscopy analysis of calcium sulfate (CS-AMX) before (**A**) and after (**B**) incubation. Phosphate (P) denoted in blue, sulfate (S) in yellow, and calcium (Ca) in red.

Counts of sulfate, phosphate, and calcium elements for each disk group were recorded via EDS to confirm the elemental mapping. Both the CS-AMX and CS-MFX disks demonstrated reductions in sulfate groups, increases in phosphate groups, and consistent calcium levels when comparing samples before and after incubation. In the elemental analysis maps, phosphate is indicated with red arrows and sulfate is indicated with blue arrows (Figure 3). Thus, in a single particle, one can easily visualize the amount of each element present (Figure 4).

**Figure 5:**
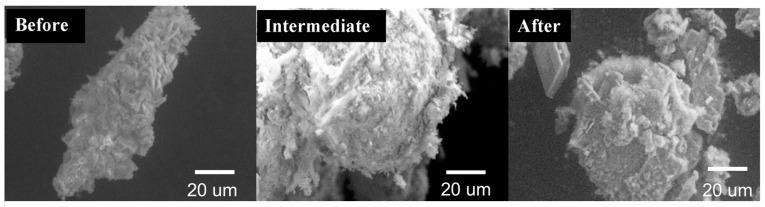
Scanning electron microscopy images showing calcium sulfate disks pre-incubation (Before), at day 30 (Intermediate), and post-incubation at day 60 (After). Calcium phosphate formation was noted on the surface of the disks at the intermediate and post-incubation stages.

Variations in the surface topography were observed with increased incubation time. SEM scans performed on the CS-AMX and CS-MFX disks before incubation revealed needle-like or plate-like crystals with sharp, well-defined edges, which were characteristic of CS [39]. After incubation (Figure 5) for 30 and 60 days, there was a marked change in the crystal shapes and surface topography. Specifically, all groups demonstrated the presence of spheroid crystals with poorly demarcated borders. This was due to the surface conversion of sulfate to phosphate, and EDS showed lower quantities of sulfate and increases in phosphate [40].

### 3.3. Antibiotic Activity and Release Profiles

**Figure 6:**
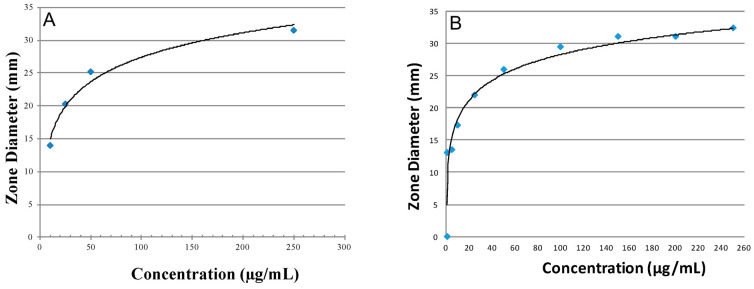
Standard curves for antibiotics, (**A**) AMX and (**B**) MFX, at their different concentrations with respect to inhibition zone sizes against *S. mutans*.

**Figure 7:**
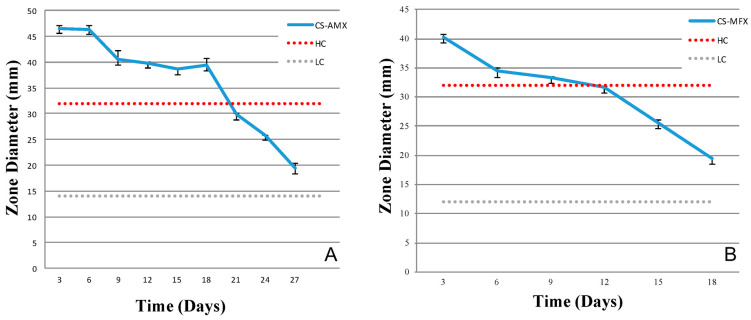
Drug release profiles in terms of antibacterial effects of the antibiotic-loaded CS disks, CS-AMX (**A**) and CS-MFX (**B**), against *S. mutans* based on diffusible inhibition zones. Antibiotic reference standard: high concentration (HC, 250 µg/mL for AMX and MXF) and low concentration (LC, 10 µg/mL for amoxicillin, 5 µg/mL for moxifloxacin).

Standard curves for both AMX and MFX, at known concentrations of the antibiotic with respect to diffusible zones of inhibition, are shown in Figure 6. The inhibition zone diameters for LC and HC for the CS-AMX disks were 15 mm and 32 mm, respectively, whereas for the CS-MFX disks, they were 11 mm and 33 mm, respectively. Both the CS-AMX and CS-MFX disks exhibited larger zones of inhibition than the set LC for the two antibiotics against *S. mutans.* Initially, the CS-AMX disks showed a higher inhibitory effect, with an inhibition zone diameter of 47 mm as compared to 40 mm observed with the CS-MFX disks (Figure 7). However, the CS-MFX disks experienced a rapid decline in potency/release beginning on day 12, whereas a similar decline occurred at day 18 for the CS-AMX disks. The CS control disks showed no inhibition zones. In addition, the CS-AMX disks remained active for 9 more days than the CS-MFX disks (Figure 7).

## 4. Discussion

Osteomyelitis can have debilitating consequences for the patient. In addition to the administration of antibiotics, particularly when chronic in nature, osteomyelitis may require large surgical resection of the bone to healthy tissue. The resulting defect, or dead space, must be filled and ultimately reconstructed with an osteoconductive material that not only repairs the defect site but can also gradually release an antibiotic in the infected site. Potential dental applications for antibiotic/graft material composite biomaterials include vascular changes associated with radiation therapy [41], osteosclerosis associated with osteomyelitis, and medication-related osteonecrosis of the jaw [42,43,44]. While polymethyl methacrylate-based products and surgical cements are the most widely used carriers for antibiotics, they do not possess osteogenic properties and can release toxic substances during curing, necessitate a secondary surgery for removal, have an exothermic polymerization sequence, and are expensive relative to ceramic-based biomaterials [45,46]. Also, the use of PMMA impregnated with antibiotics is limited by requiring heat-stable antibiotics to endure the exothermic polymerization process and removal after the cement becomes inert, thereby contributing to the often multi-staged management of these infections [47]. As an alternative to more traditional void-filling materials and grafts, this study proposes to use CS, which has bioresorbable and osteoconductive properties, and combine them with an antibiotic for the controlled release of the active antibiotic. The bacteria *S. mutans* was utilized as an indicator strain since it is fastidious, easy to cultivate, and is a primary etiological agent of many infections [48,49].

The CS disks loaded with or without antibiotic demonstrated an ability to degrade over time, which, when treating osteomyelitis, would preclude the need for a secondary intervention to remove the graft as is common with other non-degradable composite biomaterials. As the infected tissue takes 3–4 weeks to revascularize, it is in this stage that a bioactive antibiotic drug-loaded scaffold can be of most benefit [6]. The degradation rates of the disks increased with the addition of AMX or MFX and, although the difference between the CS-AMX and CS-MFX degradation rates was minimal, these composites degraded approximately 26% faster than the CS disks without an antibiotic additive. This could be attributed to the water solubility of the antibiotics since, as observed during composite mixing, the CS-AMX and CS-MFX disks required 20% more saline than the CS control disks. Furthermore, particle size has an important role in degradation, which in the case of CS disks occurs through surface erosion [50] Since AMX and MFX (<45 µm) are smaller than CS particles and 10% of the weight of the CS-AMX and CS-MFX disks came from the antibiotics, the lower of the composites could potentially accelerate their degradation [51].

Elemental mapping and surface characterization revealed the potential osteoconductivity of the CS disks. The EDS scans performed at pre- and post-incubation revealed increases in phosphate and decreases in sulfate, which could also be observed via SEM images. The SEM images showed the conversion of the pre-incubation needle-like topography, consistent with CS particles, to bulbous, oval-shapes, consistent with CaP crystals. Thus, material characterization techniques demonstrated the presence of CaP on the disk surface, which has been associated with bioactivity [52,53,54]. CaP formation occurs through the binding of phosphate ions within PBS to calcium ions released from the dissolving CS. The partial surface conversion of CS to CaP facilitates integration with surrounding bone tissue, an important indicator of the potential osteoconductivity of the CS disks [55]. Consequently, the scaffold no longer acts simply as a carrier material but also becomes a bioactive, osteoconductive bone substitute [56]. Of note, FTIR did not show this conversion because the CaP that formed on the surface of the disks was not evenly distributed. As only 1 mg of precipitate was collected for analysis, there was not enough CaP present to be detected by FTIR (Figure 2).

The addition of AMX and MFX to CS powder provided antibacterial properties to the composite disks. The antibacterial activity demonstrated through the agar diffusion assay suggested that both the CS-AMX and CS-MFX disks exhibited periods of sustained antibiotic release, predominantly days 6–12 and days 9–18 for MFX and AMF, respectively. Both AMX and MFX are heat-sensitive drugs and their activity may likely be reduced over time as they were incubated at 37 °C. Also, agar diffusion assays may not be sufficiently sensitive to detect low concentrations of the antibiotics, thereby leading to perceived inactivation below a certain concentration threshold. Despite this limitation, assay diffusion tests are efficient and can be more directly correlated with the inhibition zone and antibiotic activity than other assays [57,58].

In many cases of chronic osteomyelitis, a parenteral antibiotic therapy regimen is typically maintained for at least 4 weeks [1,59]. A 400 mg intravenous dose of MFX, for example, reaches a maximum serum concentration of 4.9 μg/mL and, given a bone bioavailability of approximately 50%, a concentration of 2.45 μg/mL at the infected site [6]. As noted in Figure 5, the eluted drug concentration from the CS-MFX disks remained well above the LC of 5 μg/mL for a period of 18 days, which was higher than both the concentration provided by parenteral dosing and the MIC against *S. mutans*. Additionally, a 2 g intravenous dose of AMX attains a maximum serum concentration of 190 μg/mL and, given a bone bioavailability of about 5–20%, a concentration of 9.5–38 μg/mL at the infected site [6,60]. The AMX concentration from the CS-AMX disks remained above the LC of 10 μg/mL beyond 18 days. While lower than that achieved by parenteral dosing, the CS-AMX disks utilized only 1% of the amoxicillin dose compared to this standard intravenous therapy; thus, a much higher local concentration may be achievable by embedding more antibiotic into the disks. Furthermore, the concentrations of the eluted antibiotics remained higher than the set HC (250 μg/mL for both) during the initial 3–6 days, which can potentially provide adequate local antibiotic coverage while parenteral therapy is initiated.

Ultimately, the antibiotic-impregnated CS disks have the potential to offer better local infection control than that achievable through parenteral antibiotics. Also, the single drug-loaded CS disks are able to elute heat-sensitive antibiotics, an advantage over other materials, such as PMMA, which require heat-stable drugs. This allows for enhanced targeted therapy and antibiotic selection based upon cultures rather than heat stability properties.

## 5. Conclusions

CS disks impregnated with antibiotics demonstrated bioresorbable, osteoconductive, and antibacterial potential for the treatment of infected bone defects, as seen in cases of osteomyelitis. As these composites degrade relatively quickly over time, future research should focus on assessing the biomechanical integrity of these ceramic disks. Developing more mechanically stable constructs capable of providing immediate structural support to critical-size defects is essential. Additionally, further in vitro studies on the cytotoxicity of the materials and osteogenic properties of the material, as well as in vivo studies involving other pathogenic microorganisms, are necessary to better evaluate the regenerative properties of CS disks. This will help to further characterize their utility and efficacy in an osteomyelitis model, ensuring a comprehensive understanding of their potential applications.

Furthermore, considering the varying degradation rates of these composites, it is imperative to explore modifications that could enhance their mechanical stability and longevity. Understanding the interactions between the ceramic matrix and different antibiotics can also lead to optimizing their antibacterial effectiveness. The ultimate goal is to create a robust, multifunctional biomaterial that not only supports bone regeneration but also effectively combats infection. This dual functionality could revolutionize the treatment protocols for osteomyelitis and other bone-related infections. Future studies should aim to integrate advanced imaging techniques and biomechanical testing to monitor the performance of these CS disks in real time. Collaborations across disciplines, including materials science, microbiology, and clinical research, will be key to achieving these advancements and translating them into clinical practice.

## Data Availability

The original contributions presented in the study are included in the article, further inquiries can be directed to the corresponding author.
